# Painless Progressive Swelling of the Foot with Discharging Sinuses (Madura Foot)

**DOI:** 10.4269/ajtmh.21-0520

**Published:** 2021-07-12

**Authors:** Rajeev Nayan Priyadarshi, Manoj Kumar, Manoj Kumar

**Affiliations:** ^1^ Department of Radiodiagnosis, All India Institute of Medical Sciences, Patna, Bihar, India;; ^2^ Department of General Surgery, All India Institute of Medical Sciences, Patna, Bihar, India

**Figure 1.**
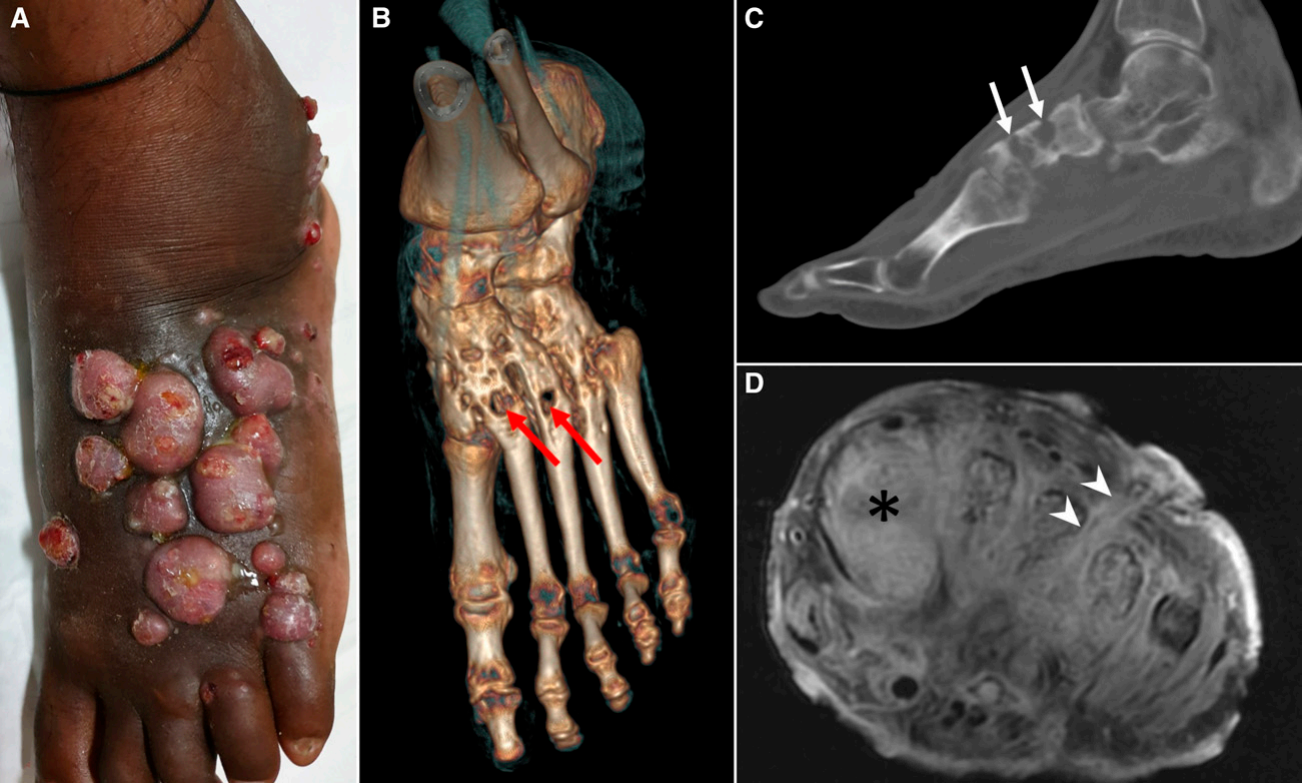
(**A**) Multiple nodular swellings with sinuses discharging serosanguineous pus over the left foot. Three-dimensional volume-rendered reformatted computed tomography (CT) image (**B**) and sagittal CT image (**C**) show multiple osteolytic lesions (arrows) in tarsal and metatarsal bones. Fat-suppressed contrast-enhanced T1-weighted magnetic resonance image (**D**) showing diffuse hyperintensity (asterisk) and enhancement involving the metatarsal bones and surrounding soft tissues. Note sinus tract denoted by arrowheads. This figure appears in color at www.ajtmh.org.

**Figure 2.**
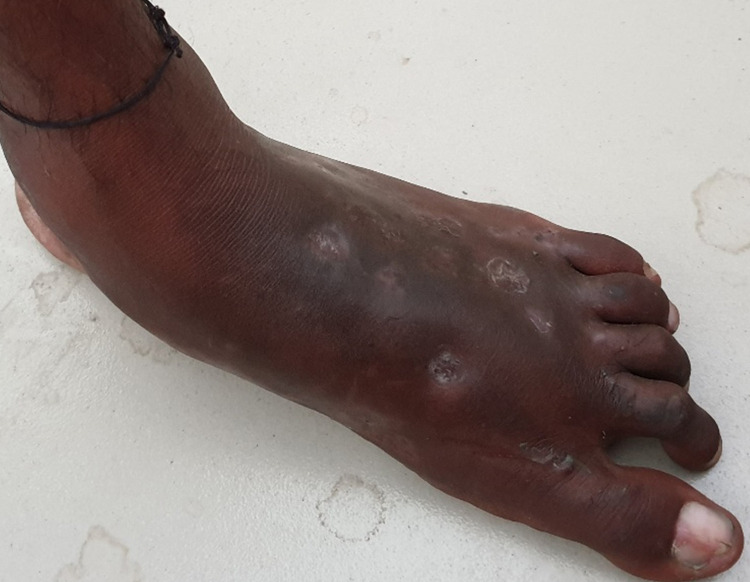
Follow-up examination at 2 months after antibiotic treatment shows complete healing of the nodular lesions with no evidence of discharging sinuses. This figure appears in color at www.ajtmh.org.

A 40-year-old man, a laborer from Bihar (eastern India), presented to the surgical department with multiple large nodular swelling and discharging sinuses over the left foot with a duration of six months. These lesions began as small nodules that progressively increased in number and size and developed multiple sinuses. On examination, the swelling was painless and firm. He had no significant medical history. Computed tomography showed extensive osteolytic destruction of the tarsal and metatarsal bones. Magnetic resonance imaging demonstrated involvement of soft tissue with multiple sinus tracts (Figure 1). Gram staining from the discharging pus revealed actinomyces, a Gram-positive filamentous bacteria. The patient received co-trimoxazole for 2 months. Despite the widespread involvement of the bones and the soft tissue, all nodules resolved and the sinuses healed without significant deformity (Figure 2).

The term Madura foot or *Mycetoma pedis* is used to describe chronic granulomatous disease caused by true fungi (eumycetoma) or filamentous bacteria (actinomycetoma).^1^^,^^2^ Both agents produce similar lesions and are characterized by the classic triad of a subcutaneous mass, sinus tract formation, and granular discharge. However, actinomycetoma is more aggressive and destructive and invades bone earlier than eumycetoma.^1^ Imaging studies are extremely helpful to delineate the extent of bone and soft tissue involvement. Microbiological diagnosis is essential because the treatment depends on the underlying etiology. Whereas eumycetoma may require surgical debridement in addition to antifungal therapy, most of actinomycetomas are successfully treated with antibiotics.^2^
